# TYPE I INTERFERON-MEDIATED NEUROINFLAMMATORY PROGRAM AND SYNAPSE LOSS IN ALZHEIMER’S DISEASE

**DOI:** 10.1093/geroni/igz038.349

**Published:** 2019-11-08

**Authors:** Ethan Roy, Baiping Wang, Hui Zheng, Wei Cao

**Affiliations:** Baylor College of Medicine, Houston, Texas, United States

## Abstract

The cytokine family type I interferon (IFN) is a major innate immune mediator extensively studied in the peripheral immune responses but largely under-investigated in AD. Previously, we established that innate immune cells readily produce IFN in response to amyloid fibrils containing nucleic acids as cofactor. Here, we investigated whether IFN pathway is associated with amyloidosis in AD brain and contributes to neuroinflammation. By systemically characterizing neuroinflammation in multiple murine AD models, we established a comprehensive core AD neuroinflammation profile that includes several key proinflammatory cytokine families, among which IFN pathway is consistently activated. When hippocampal slice culture was stimulated with different forms of amyloid fibrils, nucleic acid-containing amyloid fibrils, but not heparin-containing fibrils, potently activated IFN pathway and triggered comprehensive neuroinflammation. In addition, stereotaxic administration of IFNβ induced an immune response in the brain of wild type mice analogous to the core neuroinflammatory profile associated with Aβ pathology; whereas selective IFN receptor blockade significantly blunted the ongoing microgliosis in AD models in vivo. Furthermore, IFN promoted microglia-mediated synapse uptake from neurons, which depended on the induction of complement C3, and blockade of IFN signaling significantly abolished the pathogenic synapse loss in AD brain. Consistent with the findings in mice, we found that genes stimulated by IFN were grossly upregulated in human AD brains. Therefore, type I interferon constitutes a major pathway within the neuroinflammatory network of AD and may represent a molecular target to restrain the pathogenic inflammatory responses.

